# “I found much more joy than I ever did as a player”– a qualitative study of the emotional journey of shattered dreams and new horizons with an ACL re-rupture in young active men

**DOI:** 10.1186/s12891-025-08730-9

**Published:** 2025-05-15

**Authors:** Rebecca Hamrin Senorski, Anna Nordenholm, Emelie Dahlin, Kristian Samuelsson, Ramana Piussi, Eric Hamrin Senorski

**Affiliations:** 1 Sportrehab, Sport Medicine Clinic, Gothenburg, Sweden; 2 Sahlgrenska Sports Medicine Center, Gothenburg, Sweden; 3 Department of Health and Rehabilitation, Institute of Neuroscience and Physiology, Sahlgrenska Academy, University of Gothenburg, Gothenburg, Sweden; 4 Elitrehab, Gibraltargatan 39, Gothenburg, Sweden; 5 Department of Orthopaedics, Institute of Clinical Sciences, Sahlgrenska Academy, University of Gothenburg, Gothenburg, Sweden

**Keywords:** Anterior cruciate ligament, Second ACL injury, Return to sport, Rehabilitation, Re-injury, Qualitative research, Interview

## Abstract

**Background:**

Ipsilateral second anterior cruciate ligament (ACL) injuries, are more common in males compared to females. An ACL re-rupture can imply both physical and psychological consequences for patients, with reports of lower objective and subjective outcomes after a re-rupture compared with primary ACL injury. This study aimed to explore young men’s experiences of their first ACL injury, reconstruction and subsequent ACL re-rupture.

**Methods:**

Eighteen males (19–33 years old) were interviewed with semi-structured interviews, which were recorded, transcribed, and analysed with qualitative content analysis, based on the method described by Graneheim and Lundman. The method involves a systematic process where three authors were involved. The interview transcripts were red multiple times, meaning units were identified, and codes were generated. These codes were then grouped into sub-categories and further sorted into main categories. Results were presented as a positive or negative psychological response through the four domains (cognition, affect, behaviour, and outcome) used in the model by Weise-Bjornsdal´s: cognition, affect, behavior and outcome, i.e., *“the model of psychological response”*.

**Results:**

Fifteen main categories and 34 sub-categories emerged the data, presented as 2 timelines between the “first ACL injury” to “ACL re-rupture” and from “ACL re-rupture” and onwards. Positive emotions with *the first ACL injury* were high motivation to return to sport (RTS), great trust in the process of recovery and great support system from friends and family. Negatively, patients expressed shattered sport dreams, physical and mental trauma leading to experiences of identity loss, and a sense of loneliness. Positive experiences with the *ACL re-rupture* were humbleness and joy for other shapes of sport participation, such as coaching, and for activity to be joyful rather than performance connected. Negatively, patients searched for answers, e.g., whether surgery and rehabilitation were performed appropriately the first time, or whether the outcome could have been different with another autograft or if RTS was delayed.

**Conclusion:**

After the first ACL injury, patients predominantly faced negative emotions that questioned their self-image. Despite several challenges, motivation remained high as the goal was RTS. Upon the ACL re-rupture, patients experienced a shift in perspective with sport participation and a door opening to new opportunities in life e.g., coaching. This unexpected turn of events became a catalyst for exploration of different aspects of emotions with the ACL journey, which included broader horizons beyond confines of athleticism.

**Clinical trial number:**

Not applicable.

**Supplementary Information:**

The online version contains supplementary material available at 10.1186/s12891-025-08730-9.

## Introduction

An anterior cruciate ligament (ACL) injury is a common sports-related injury [1]. The psychological response to an injury can be cognitive, emotional, and behavioural, and lead to psychological impairments. The athletes goals of sports participation, and the stress response from the injury may be influenced by different psychosocial factors, e.g., history of stress, personality characteristics and coping recourses [2]. The interplay of these psychological factors regulate the magnitude of the psychological response to an injury.

An ACL injury is treated with rehabilitation, with or without reconstruction of the injured ligament [3]. A reconstruction of the injured ligament is often recommended for patients who aim to return to pivoting sports [4]. After reconstruction patients have reported uncertainty of full recovery, often seen as a barrier for not resuming pre-injury activity [5], and fear towards the risk for a second ACL injury [6]. 

Up to 14% of patients who are treated with ACL reconstruction suffer a secondary ipsilateral ACL injury i.e., a re-rupture [7], which can imply both physical and psychological consequences for a patient, where lower objective and subjective outcomes are reported after re-rupture compared with primary ACL injury [8–11]. Several risk factors for a re-rupture are known, and include sex, where contralateral injuries are reported higher among females while re-ruptures are slightly higher in males [12], young age, were people < 25 years old are at higher risk than patients > 25 years old [13, 14], high activity level, graft type [15], as well as the use of an extra-articular tenodesis or not [16]. Moreover, a return to sports (RTS) < 9 months has been associated with an increased rate of ACL re-rupture [17]. Even though males report a greater re-rupture rate they seem to have a more positive outlook on their knee-related self-efficacy compared to females [18]. 

To integrate qualitative research, and patients’ own experiences into the scientific knowledge could aid researchers and clinicians to understand the burden of an ACL re-rupture. A qualitative study that investigated young females’ perspective on an ACL re-rupture has already been completed, and results show no positive emotions after the primary ACL injury, although both positive emotions and personal growth with the re-rupture were reported [19]. How young men experiences an ACL re-rupture is however not well studied and needs further investigation. This study aimed to explore young men’s experiences of their first ACL injury, reconstruction and subsequent ACL re-rupture.

## Materials and methods

### Study design and participants

This study was conducted as a qualitative interview study, with data based on semi-structured interviews to answer the research question. A deductive approach was adopted, where the results were organized according to the domains in the “integrated model of psychological response” described by Wiese-Bjornsdal [20]. For transparency in the methodology, the Consolidated criteria for Reporting Qualitative research (COREQ) [21] checklist was used. An interview guide was created by authors RP and EHS through extensive discussion and screening of the literature on the subject. Patients that participated in the present study were recruited from the rehabilitation registry Project ACL, started 2014 in Gothenburg, Sweden. A purposive sample of patients registered in Project ACL, aged between 15 and 35 years, who reported an ipsilateral second ACL injury (re-rupture) were selected from the registry. Authors ED and RP contacted patients to confirm the occurrence of an ACL re-rupture. Subsequently, patients were informed of the study and asked whether they were interested in participation. Upon a positive response, a meeting was scheduled. Prior to agreeing to participate in the study, all patients were informed that participation was voluntary and that withdrawal from the study with retraction of the interview was possible at any time. None of the contacted patients declined participation. At the outset of each interview informed consent was confirmed orally and recorded. The study was performed in accordance with the declaration of Helsinki, ethical approval was obtained from the Swedish Ethical Review Authority (registration number 2020–02501).

To ensure reflexivity, the interaction between researchers and the data, the authors background should be reported: the first author (RHS) is a female physical therapist and PhD student with 8 years of experience in ACL rehabilitation, who has sustained an ACL injury during sports participation, treated with reconstruction. The second author (AN) is a female physical therapist with a PhD from 2021, currently working clinically. The third author (ED) is a female physical therapist with an additional Degree of Master of Science in Medicine, with experience in sports physical therapy. The fourth author (KS) is a male orthopaedic surgeon and professor, who has sustained an ACL injury to each knee, primarily working with patients who suffer an ACL injury in need of a reconstructive surgery. The fifth author (RP) is a male physical therapist and PhD, with experience in qualitative research, and nearly a decade of experience with sports rehabilitation for patients with an ACL injury. The senior author (EHS) is a male associate professor with several years of experience in ACL rehabilitation and research. Author EHS is currently also the senior physical therapist at a sports rehabilitation clinic. None of the authors had any prior clinical, professional, or personal relationships with the patients included in the study. The idea of this study was generated through a previous study on young female patients, published in 2022 [19]. 

### Data collection

Eighteen patients were contacted, and since no patient declined participation, 18 individual semi-structured interviews were conducted. Due to the rich and broad information gained under the first 14 interviews, no new information emerged after them; thus, authors feel confident that the data collected was sufficient to answer the study question. An additional 4 interviews were performed to ensure that no new information emerged, and consequently, 18 interviews in total were performed in this study. All statements from the patients were analysed confidentially. All interviews were carried out by authors ED and RP. Eighteen active male patients were included in the study, with a mean age of 26.8 (range 19–33) years at the time of inclusion and a mean of 26.1 months (range 6–96) between primary ACL reconstruction and re-rupture (missing *n* = 1). At time of inclusion, most patients were active in football, handball, and skiing, as presented in Table 1.

**Table 1:** Injury history of included patients

Patient	Age (years)	Main sport (at time of injury)	Return to training with restrictions (months)	Return to unrestricted sports (months)	ACL re-rupture (months)	Time of interview (months)	Treated with revision
1.	19	Football	5	8	9	18	Yes
2.	27	MMA	7	10	36	42	Yes
3.	21	Handball	8	12	18	27	Yes
4.	29	Football	9	11	96	114	Yes
5.	22	Football	9	12	36	90	Yes
6.	27	Football	7	n/a	96	144	Yes
7.	24	Volleyball	13	n/a	13	66	Yes
8.	26	Football	5	n/a	6	66	Yes
9.	31	Skiing	8	11	11	72	Yes
10.	32	Football	4	11	13	54	Yes
11.	24	Alpine skiing	18	n/a	48	54	Yes
12.	29	Football	9	n/a	10	84	Yes
13.	27	Floorball	n/a	n/a	6	90	Yes
14.	28	Football	6	12	12	102	Yes
15.	26	Handball	12	14	14	108	Yes
16.	33	Football	7	n/a	9	120	Yes
17.	30	Football	9	11	11	110	Yes
18.	27	Football	n/a	n/a	5	120	Yes

ACL, Anterior Cruciate Ligament; n/a, not applicable– the patient did not reach this step prior to re-rupture; MMA, mixed martial arts

Data was collected between February and April 2023. Interviews were performed digitally (via ZOOM, web-based application) using open-ended questions such as, “How did the injury and rehabilitation affect you?”, or “How did you feel going through rehabilitation once again?”. The interview guide can be found in Appendix Table 1. No other person beside the interviewer and the patient were present at the time of the interview. During the interviews, no field notes were taken, and the author that performed the interview did not provide their own biases or assumptions, in order not to bias patients, and to collect their unaffected experience. Interviews were audio recorded, and the records were transcribed verbatim. Transcripts were not sent to participants for corrections or comments to preserve authenticity, and due to criticism of the method of interviewee transcript review which could produce substantial alteration to the data [22, 23]. 

### Data analysis

**Fig. 1:**
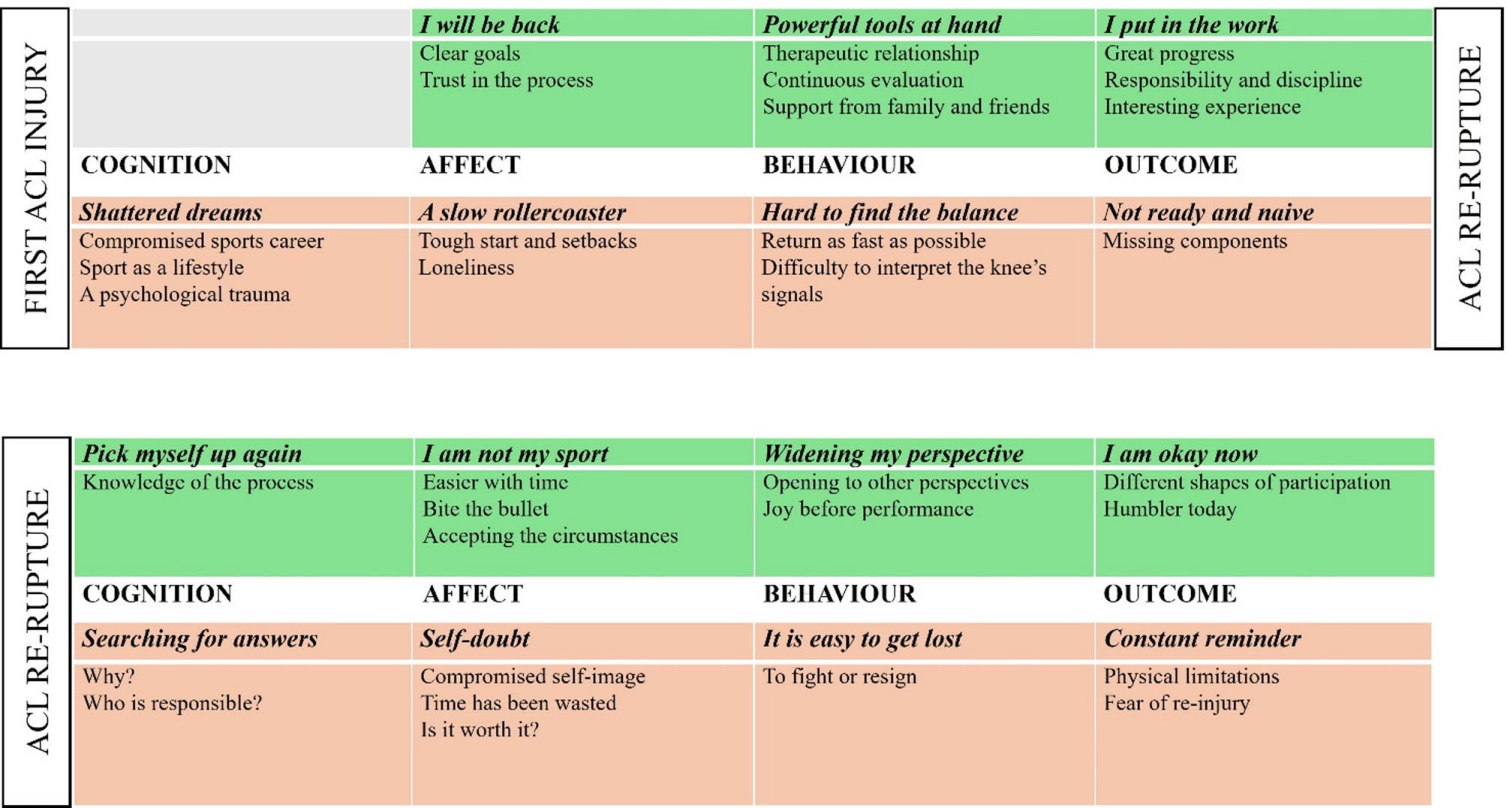
Extracted main categories (italic bold) and sub-categories (below each main category) based on Weise-Bjornsdal´s model

Data was analysed using qualitative content analysis based on the description of Graneheim and Lundman [24, 25]. Results were then presented according to the integrated model of psychological response used in the deductive approach for the data analysis. The integrated model of psychological response was originally presented as a circle, which means that psychological response to a sports injury is cyclic and components are inter-related [20, 26]. We chose to present the results divided into positive or negative psychological response for each of the four domains used in the model by Weise-Bjornsdal´s: cognition, affect, behavior and outcome [20] in two different timelines, the first timeline representing the time from first ACL injury to the ACL re-rupture and the other from the ACL re-rupture to the time of the interview (Fig. 1).

Authors RHS, AN and ED were responsible for data analysis with a continuous dialog with authors RP and EHS. Firstly, the transcribed interviews were thoroughly read in entirety several times by authors RHS, AN and ED to gain a comprehensive overview of the interview texts. Secondly, meaning units, that is, sentences that relate to the same central meaning which relate to each other through content and context were extracted into Microsoft Excel (Microsoft Corporation, Redmond, WA, USA) by authors RHS, AN and ED. The meaning units were then condensed in the same Excel sheet by the same three authors (RHS, AN, and ED). Thirdly, the extracted meaning units were abstracted into codes by authors RHS, AN and ED. Fourthly, codes were categorized, where codes that addressed similar categories were grouped into sub-categories by authors RHS and AN. The codes included under each sub-category were continuously read and triangulated between authors RHS and AN to ensure codes were not erroneously grouped. Upon disagreement or if the authors could not decide or place a code under a sub-category, authors RP and EHS were consulted for assistance. Transcribed interviews were continually read to ensure that data was appropriately understood in relation to the context of the study. Fifth, when agreement was settled, the sub-categories were then grouped into main categories by authors RHS and AN. The main, and sub-categories were then sorted into the different domains of the integrated model of psychological response [20] and polarised as positive or negative. The process was performed firstly for the timeline between the first ACL injury up to ACL re-rupture, and then secondly for the timeline between ACL re-rupture and the time of the interview.

## Results

Out of the 18 patients included, 2 patients (11%) never return to training prior to their re-rupture and an additional 6 (33%) never return to unrestricted sports prior to re-rupture. Fifteen main categories and 34 sub-categories emerged from qualitative content analysis. The visual representation of results from this qualitative study were divided into 2 timelines, the first from the first ACL injury to ACL re-rupture, and the second from ACL re-rupture to the time of interviews, presented in Fig. 1.

## The first ACL injury

The first timeline, ACL injury sequence (Fig. 1) represents the journey from ACL injury to the ACL re-rupture where patients go through the first rehabilitation process and experiences for the first time. Patients experienced high motivation for RTS and expressed trust in the process of rehabilitation. Support from family and friends as well as the continuous evaluation of muscle function helped as a guidance in rehabilitation. However, patients expressed that their dreams with sports got shattered. The ACL injury was perceived as a mental trauma, which led patients to experience a loss of identity and wonder who they were without their sport. Patients who RTS also experienced that RTS happened too quickly and wished someone had helped them not to stress the RTS. In hindsight, patients felt naive to have RTS not feeling completely ready.

### Cognition

The patients experienced *“shattered dreams”* (main category) after their ACL injury.

I had a contract from a larger club on the table and /…/ an offer to go to England and see clubs there /…/ but I knew it was all gone when I got the message (of the ACL injury) /…/ It was /…/ really tough (p12)

Furthermore, patients expressed that the ACL injury was not only a physical trauma to the knee but also a psychological trauma. Some patients expressed that perceived lack of sufficient help from the health care system with the mental stress due to the knee injury.

I wished the healthcare system had helped out more with the mental aspect after the injury as I felt bad mentally too /…/ that you should not dwell on the accident /…/ For me it was a lot of thoughts. (p7)

The patients did not report any positive experience associated with the ACL injury.

### Affect

Patients were positive to the rehabilitation process and were sure their effort would result in RTS, which was explained with the category *“I will be back”.*

I got kind of a new goal /…/ and then it was just to get back and play again /…/ so its /…/ I don’t know I shook it off pretty fast (p18)

Patients stated that they trusted the process of rehabilitation and explained that clear goals in training helped with motivation, as did assistance with goal setting from the treating physical therapist.

My physical therapist was very good at putting together a plan and schedule for what I was supposed to do and when I was supposed to do it /…/ we also tried new things based on how the tests went. (p8)

As a negative response, the rehabilitation process could be experienced as slow and fraught with setbacks such as increased pain, swelling, decreased mobility. Patients expressed a hard time to understand the pain and a lack of help to interpret the pain.

Pain has been the hardest thing /…/ how much pain is okay how much pain am I supposed to have /…/ it is hard to know all the time /…/ is it necessary pain and where is the pain coming from (i.e. anatomical structure) /…/ if I have done something wrong /…/ personally I would have needed more help to distinguish it or someone who either had stopped or pushed me (p13)

With setbacks and absence from sports the patients experienced a sense of loneliness. Occasionally the patients felt self-doubt, and wondered if they would ever RTS.

You felt very alone /…/ you were in rehabilitation yourself /…/ if you play football /…/ you are in a larger group and you train together and you do things together /…/ whereas now it turned out that everyone else did it and I had to train alone to rehabilitate my knee. (p1)

### Behavior

The therapeutic relationship with the physical therapist, continuous evaluation, and support from family and friends together constituted to the category *“powerful tools at hand”.*

The whole family is behind you and friends were /…/ happy to help (p9)

It was easy to follow the physical therapists program since it was very clear and I had so much trust (i.e. in the physical therapist) since I knew he was an expert and I just trusted him 100% (p14)

The continuous evaluation made the patients more confident in the process of RTS, which also kept the motivation high.

Every time you felt that there had been an improvement /…/ you could handle another kilometer or so /…/ every time you saw an improvement it felt extra good because then what you had been fighting for had paid off. (p17)

However, patients experienced it was *“hard to find the balance”* in the final stages of rehabilitation after the first ACL injury and expressed difficulties with how to interpret knee signals in terms of e.g. increased pain or swelling.

It is a challenge to progress exercises /…/ it is like the body is working against me a bit /…/ I feel like in terms of strength I can push myself more, but the pain is the thing holding me back /…/ and that it so easily gets swollen (p9)

Furthermore, patients faced a certain degree of pressure to RTS even though they did not feel completely ready. Despite that muscle function was perceived as recovered, patients felt a mental block, with insufficient confidence in the knee´s ability to tolerate and withstand the load of cutting and pivoting activity. In retrospect, the patients wished that someone held them back, or stopped them from an early RTS.

…going back too early /…/ although, they (i.e. the physical therapist) had said that it was not too early but I still had that feeling. (p1)

### Outcome

In the category *“I put in the work”*, patients experienced a great recovery process after the first ACL injury.

So that have certainly made it easier for me to carry out tasks that I do not find fun now then because /…/ you have taught yourself and maybe fool yourself to think that it (rehabilitation) is reasonably fun, although it may not really feel that way. (p6)

The patients stated that the closer they came to RTS, the harder it was to maintain discipline in rehabilitation, as increased on-field training limited time at the gym.

You did not really notice any symptoms but still needed to motivate yourself to continue doing the exercises to not injure yourself again /…/ and most likely I loaded too hard too soon and that’s why it ruptured again (p6)

As summarized in the category *“Not ready and naive”*, patients did not feel ready for RTS and expressed a worry for excessive forces applied on the knee. Patients returned to sport anyway since they were cleared for RTS by the responsible physical therapist.

I kind of had no strength to resist the rotational violence because there was none /…/ not close to the muscle strength on the back of the thigh that I have on the other leg when it’s healthy. (p7)

All patients went on to sustain an ACL re-rupture.

## The ACL re-rupture

The ACL re-rupture sequence (Fig. 1) represents the journey from the ACL re-rupture to the date of interview. Patients experienced a new humbleness and accepted that sport does not define a person´s identity. Also, participation in sports can have many shapes, such as coaching, and not only self-participation. Patients also expressed a desire for activity to be more joyful than connected to performance. However, patients who RTS expressed wondering if rehabilitation and their effort were all worth to RTS or if the time elapsed and the effort put in to rehabilitation was just a waste since the ACL was re-injured anyway.

### Cognition

After the ACL re-rupture, patients found themselves with ambivalent feelings about the process to come. They expressed a sense of calm based on knowledge about the process to come, yet unease about the length and work of the process, and sadness to go through the same procedure again. However, the calm overshadowed the uncertainty, and patients expressed it was just to push on, which resulted in the main category; *“pick myself up again”.*

… it was tough mentally again because then you are also aware of the rehabilitation process /…/ how long it is and how demanding it is /…/ so it was nice to be aware of what I had in front of me as well /…/ so it was probably a bit of both. (p7)

With mixed feelings about the process to come, patients felt a need to understand why the ACL re-rupture happened. In this search for understanding, patients started to *“search for answers”* which shaped this main category.

I felt like he (the physical therapist) saw me as a failure, that I didn’t do well /…/ but it was on his and others initiative /…/ that I increased my training all the time and the doctor said it was his (the physical therapist) fault that I shouldn’t have been running on artificial grass /…/ and the physical therapist said that the doctors must have done something bad in the operation. /…/ They just blamed each other but really I´m the one who’s affected and had to pay for it. /…/ You always want to blame someone or something in order to find some explanation. (p13)

Patients meant that they were cleared for RTS based on the physical aspects, but were never asked about their mental status, and sometimes felt the mental preparation for return was overruled of the physical aspect. Patients also missed the support from a psychologist which patients determined as essential to process the trauma of an ACL injury and to rebuild trust in the knee.

And then, above all, to talk to a therapist or a psychologist or a life coach or something like that about how to process /…/ a physical trauma. (p7)

### Affect

Patients experienced negative emotions with the ACL re-rupture eased over time. Patients realized that other aspects of life might be more important, such as being able to run in the future, and to participate in activities with friends. The clear drive and determination to RTS after the first ACL injury was no longer as absolute.

I no longer identify myself as strongly with the sport /…/ that I’m not handball and handball isn’t me and I do not value myself in the same way as I did on the court (p8)

Patients felt hopelessness, grounded in self-doubt, which led them to wonder if the struggle with physical function and mental stress was worth RTS, just to re-injure the ACL again.

You have had to change your self-image a little that you might not be as physically strong as you might have /…/ wanted or as you think you are /…/ that even if you were happy with the result, maybe you should have done a little more. (p12)

In reflection to the previous rehabilitation, patients expressed a conflict on whether it was worth to push for RTS if the result was an ACL re-rupture.

It has certainly put some mental barriers around what I think I can /…/ I have landed in a realization that I /…/ get to choose what I want to do if it’s worth the risk. /…/ Well, I don’t know if I do, because I think there is so many other sports that are fun and I have no desire to injure the ACL a third time. (p17)

### Behavior

Under rehabilitation, patients expressed to have *“widened their perspective”* in terms of what the sport meant to them. The strive for sports participation could instead take other shapes or forms such as happiness through coaching sports for instance.

I would not have wanted to rupture my ACL the second time, but at the same time, then I would never have become a coach as early as I did. Because when I ruptured the ACL the second time my club wanted me to become a coach for the juniors and /…/ I found much more joy than I ever did as a player (p1)

Patients also expressed it was “*easy to get lost”* in the expectations of continuity in rehabilitation and the ambition of RTS.

I felt I did not have the energy for it (rehabilitation again) /…/ After a while I tried to rehabilitate but I just felt no, I cannot take it. (p11)

The responsibility for load monitoring rested with the physical therapist as the expert. When the load exceeded a manageable threshold, patients experienced frustration in load management.

I would suspect that there was simply too much volume. I do not think the exercises were too complicated or too hard on the knee. I just think it was just too much. (p8)

### Outcome

At the culmination of the recovery journey after an ACL re-rupture, patients expressed a sense of humility, accompanied by decreased concern about the type of physical activity carried out, and reflected on what might have been without the injuries. Patients attained a level of calmness regarding their current knee function, despite their initial reluctance to experience the ACL injury.

I think I have accepted pretty much what /…/ I can do and not

Patients no longer felt rushed to get back to a specific type of training or sport but would rather want the knee to tolerate the load applied in daily life. The patients expressed they could better understand the knee’s boundaries and could more easily consider the consequences regarding the knee’s function than before the ACL injury.

Right now, it is good (the feeling) /…/ I train football in that amount that I can /…/ it is easier for me to sense when the body is tired and /…/ time to quit that practice /…/ even though you wish you had never gotten injured in the first place (p6)

Though, patients explained that the knee is a *“constant reminder”* of what has been and what could have been.

The idea and intriguing feeling of being able to explore how far you can go in football, it was there then, it is not there now. But I would not say I am in a worse place in life today. (p5)

The patients expressed persistent physical and psychological limitations which infer risk assessment prior to physical activity. Patients choose to limit physical activity due to fear of re-injury instead of pushing for a second return.

It makes you think about what you do /…/ it doesn´t really stop me from anything in my daily life today /…/ more that I have to think before I do something if it is really worth it (p14)

## Discussion

The main findings of this study were that male patients who suffer an ACL re-rupture experienced both negative and positive emotions with both the first ACL injury, and the ACL re-rupture. The first ACL injury was perceived as predominantly negative, whereas the ACL re-rupture resulted in a sense of acceptance. The shift between dominating negative responses from the first ACL injury to mostly positive emotions with the re-rupture allowed patients to find more joy in the varied forms of sports participation the ACL journey provided, a joy that surpassed what sports participation itself provided. The main categories identified in this study were interpreted as positive and negative response to the specific event, based on Weise-Bjornsdal´s model [20]. We recognize that positive and negative responses may coexist within the same context and may interact with each other in a dynamic relationship.

There have been several qualitative studies performed on the experiences of ACL injury or reconstruction in female patients [19, 27, 28]. This study is unique to explore male patients experience after an ACL injury, reconstruction and subsequent ACL re-rupture after ACL injury. A similar study was conducted on female patients, that showed an ACL re-rupture resulted in “a stronger me” [19]. Male patients in the present study found more joy in another form of sport participation rather than participating in the sport themselves, as e.g., a coach.

The first ACL injury was perceived as mostly negative with affect at time of ACL injury but also for future sport participation in school or professionally i.e., due to absent draft for a greater team, or carrier ending. The ACL injury made patients unable to participate in their desired activity which led them to feel lonely. An inability to participate in social engagement can make patients change their self-image, and lead to a loss of identity [29]. Results from an interview study based on young female patients, experiences of the first journey after their primary ACL injury involved a loss of identity, a sense of loneliness and, not to be ready for RTS at time of RTS [19]. Even though patients did not feel ready for RTS, they experienced high motivation towards RTS and expressed the importance of physical therapists in the whole rehabilitation process [19]. Patients in the present study did, despite a sense of devastation due to the primary ACL injury, have a high motivation and trust for the rehabilitation process to come. The relationship with the physical therapist was perceived as important and continuous evaluation of muscle strength and function was perceived as motivational. The relationship with the physical therapist has previously been referred to as the therapeutic alliance [30]. In this alliance, it is specifically important to value emotions and perspectives with the patients through engagement in the patients rehabilitation and to have good communication. An engagement patients in the present study valued greatly, even though the psychological preparation for RTS was not optimal. Previous studies state that low psychological preparation to RTS can increase the risk for re-rupture [31], and that fear and anxiety can affect rehabilitation outcomes [32]. Patients in the present study experienced feeling rushed for RTS under the first rehabilitation although not feeling 100% ready, especially mentally. Physical therapists should especially consider patients mental preparedness for RTS and not solely rely on the muscle function tests prior to RTS. If needed, a sports psychologist should be counselled to further help the patients with the mental trauma of an ACL injury and prepare patients mentally for RTS. Patients described a struggle to interpret the knees signals such as increased swelling or pain, which highlights the need to equip patients with enough knowledge on load management, and how to manage symptoms on a daily basis, depending on rehabilitation phase and the general knee status [33]. 

The ACL re-rupture was perceived as devastating, where patients tried to find someone to blame, and questioned if the choice of autograft could have been the reason for sustaining the ACL re-rupture. There are pros and cons with all autografts, even if patients wondered why the second ACL reconstruction autograft (believed to be “stronger”) was not chosen for the primary reconstruction. Previous studies on female patients have shown that an ACL re-rupture is perceived as a lifelong adaptive cooping process [27, 34], and that persistent fear of re-rupture has been described to result in long-term lifestyle modifications [35]. Patients in the present study also expressed to have adapted the physical activity level to their knee capacity. The physical activity level adaptation opened another view of participation, e.g., to become a coach. Patients also expressed that training with friends, and to have the possibility to do social sports activity overweighted the negative emotions of the decision of not RTS again. After accepting not returning to sport, the patients felt humbler, however, still experiencing a persistent physical limitation. Despite a lower activity level and change of participation focus, patients experienced their knee as a constant reminder as long as 9 years after ACL re-rupture. The study by Heijne et al. [27], also suggested that patients accepted their new lifestyle as time went by. However, the male patients in the present study had widened their perspective and stated to be okay now, which can imply patients have come to terms with another lifestyle, despite not expressing acceptance towards the new perspective.

If there was something that the patients would have done differently, patients wished someone had stopped them from RTS so early in rehabilitation. They also wished to see a psychologist, to gain help with processing the mental trauma that an ACL injury entails. Patients also wanted the psychologists support with the psychological preparation for RTS which the physical therapist could not entirely provide. In summary, psychological factors should have a greater place in the rehabilitation after an ACL injury as patients experience both a physical and psychological trauma with an ACL injury. The support system should involve family and friends, but also physical therapists and psychologists to prepare the patients for a potential RTS.

### Methodological discussion

A qualitative content analysis based on Graneheim and Lundman [24, 25], with a deductive approach presented as positive and negative response trough “the integrated model of psychological response” by Weise-Bjornsdal [20], was used in the present study. Furthermore, response can be spiraling, and different responses (affect/behavior/outcome) can occur at the same time, but for pedagogic purposes we chose to plot them as a timeline from primary ACL injury to re-rupture and from ACL re-rupture onwards. Qualitative content analysis allows analysis of different experiences, perceptions and realities within the same population and was therefore determined as a suitable method of choice. Furthermore, 18 interviews were conducted for the present study. No further information emerged after the first 14 interviews. We therefore feel positive that we have captured the patients’ full experiences of a first ACL injury and ACL re-rupture. Trustworthiness in the present study was ensured by three concepts: credibility, dependability, and transferability. Credibility refers to how well the data represents the focus of the study. The cohort consist of male patients active in different sports, at different ages who have different times between RTS and ACL re-injury. The diversion in the cohort allowed us to capture many aspects in the same cohort. Dependability refers to the degree to which the data changes over the analysis process and over time. Dependability was considered and ensured as no changes were made to the interview guide under the interview process. The interviews were held within a couple of weeks, and analyses were made short after between three authors (RS, AN, ED) with a continuous dialog with the fifth (RP) and senior (EHS) authors. Furthermore, triangulation was performed gradually through the entire process with researchers with high experience in qualitative content analysis, which include experts in sports physical therapy. Transferability refers to the possibility to transfer the results of this study to other settings or other groups. Transferability is not always the goal with qualitative research and should be judged by the reader.

### Limitations

There are some limitations with the present study. Firstly, patients were recruited from the rehabilitation registry Project ACL. Patients included in Project ACL are regularly evaluated with muscle function tests and patient reported outcomes which can contribute as a motivational booster. Thereby, patients included in the present study might have a higher motivation to rehabilitation or RTS compared to the general ACL population. However, the study does not specify whether there were standardized rehabilitation protocols followed by all participants. Differences in rehabilitation approaches could influence the outcomes and experiences reported by the participants. Furthermore, differences in opinion between orthopaedic surgeons, doctors, physical therapists, coaches, strength and conditioning coaches might confuse patients in what to focus on and how to reach set goals in a sufficient way. Patients were recruited from a single geographic area (Gothenburg, Sweden). This may limit the transferability of the findings to other regions with different healthcare systems, rehabilitation protocols, or cultural attitudes toward sports and injury. Furthermore, while the study includes 18 participants, no further information emerged after 14 interviews. Thereby, authors are confident the number of interviews were sufficient to elucidate adequate emotions associated with the ACL injury journey. The sample also consists only of male patients who have experienced an ACL re-rupture, which is merely a subgroup of patients who sustain an ACL injury. Another limitation is the possible risk of recall bias. The study relies on participants’ recollection of their experiences, which may introduce recall bias. Participants might not accurately remember or articulate their past experiences and feelings, especially if significant time has passed since the injuries and treatments, since up to 12 years had passed between the first ACL injury and the interviews. In addition, the study does not consider the socioeconomic status of participants, which could impact access to resources, quality of care, and overall recovery experiences. Socioeconomic factors might play a significant role in rehabilitation success and RTS outcomes.

## Conclusion

After sustaining a primary ACL injury male patients faced predominantly negative emotions, including loss of identity, a sense of loneliness and not feeling ready for RTS. An ACL re-rupture has, however, a positive component and can open doors for future sport participation even without own performance. Clinicians should continue with emotional support throughout the rehabilitation process and should consider the psychological recovery prior to RTS. After the first ACL injury, patients faced predominantly negative emotions that questioned their self-image. Despite several challenges, motivation remained high as patients actively pursued rehabilitation with the ultimate goal to return to sport. Upon the ACL re-rupture, patients experienced a shift in perspective. The re-rupture was not seen only as a setback but as a door opener to new opportunities in life such as coaching. This unexpected turn of events became a catalyst for the exploration of a different polarization of the ACL injury journey, seen not only as negative but as an opportunity to broaden horizons beyond the confines of athleticism.

## Electronic supplementary material

Below is the link to the electronic supplementary material.

Supplementary material 1

## Data Availability

The datasets used and/or analysed during the current study are available from the corresponding author on reasonable request.

## Abbreviations

ACL: Anterior Cruciate Ligament
BMI: Body Mass Index
BPTB: Bony Patellar Tendon Bone
Cm: Centimeters
COREQ: Consolidated criteria for Reporting Qualitative research
e.g.: Exempli gratia– for example
HT: Hamstrings Tendon
i.e.: id est– that is
kg: Kilograms
MD: Medical Degree
n: Number of patients
n/a: Not applicable
RTS: Return To Sport
QT: Quadriceps Tendon
