# Supplementary material for: “I found much more joy than I ever did as a player”– a qualitative study of the emotional journey of shattered dreams and new horizons with an ACL re-rupture in young active men
Source: BMC Musculoskelet Disord. 2025 May 15;26:482. doi: 10.1186/s12891-025-08730-9 (PMC12080000; doi:10.1186/s12891-025-08730-9)
Supplement: Supplementary file 1 — Supplementary material 1 [file 12891_2025_8730_MOESM1_ESM.docx]

| ***Appendix Table 1: Interview guide*** |
| --- |
| *Sports and leisure time* |
| Do you perform any sport today? |
| What does your sport mean for you? |
| What are your dreams with your sport participation? |
| What do you do besides sport (work/study)? |
| Has your ACL injury influenced your choices of work? |
|  |
| *First injury/rehabilitation* |
| How did you injure your ACL the first time? |
| How was the rehabilitation after the first surgery? |
| Do you recall anything particularly tough during rehabilitation? |
| Do you recall anything particularly easy during rehabilitation? |
| Was there anything that made you particularly sad during rehabilitation? |
| Was there anything that made you particularly happy during rehabilitation? |
| Did you get the support you wished during rehabilitation, and if so, from whom? |
|  |
| *Second injury/rehabilitation* |
| Can describe how you injured your ACL the second time? |
| How did you feel? |
| Did you directly know it was the ACL again? |
| How did you feel upon receiving the medical notification that your ACL was ruptured again? |
| Did you feel anything was missed during your treatment – if so, what? |
| What did you feel about having to go through another rehabilitation process? |
| How do you think rehabilitation has affected you as a person? |
| How do you think suffering two ACL injuries as affected you as a person? |
| How did you look at the future before the ACL re-rupture? |
| How did the ACL re-rupture affect the way in which you looked at the future? |
| How did the ACL re-rupture affect your ambitions? |
|  |
| *Social life/personality* |
| How do you think your ACL injuries have affected your social life? |
| What kind of support have you had along the way? |
| What did your teammates mean to you? |
| Have you been spending less time with your teammates because of the ACL injuries? If so, why? |
| Do you feel lonely? If so, why? |
| How has the ACL injuries affected your self-image? |
| Is there anything related to your knee that you are afraid of? |
| If you could go back in time, what would you change? |
| How do you feel today? |
| Do you have any proposition for improvement? |
| Is there anything you would like to add? |
